# Transperineal Ultrasound Before and After Prostatectomy: Technical Approach and Description

**DOI:** 10.1002/jum.16064

**Published:** 2022-07-21

**Authors:** Anna Colarieti, Nadeem Shaida, Nikesh Thiruchelvam, Tristan Barrett

**Affiliations:** ^1^ Unit of Radiology IRCCS Policlinico San Donato Milan Italy; ^2^ Department of Radiology, Addenbrooke's Hospital University of Cambridge Cambridge UK; ^3^ Department of Urology, Addenbrooke's Hospital University of Cambridge Cambridge UK; ^4^ CamPARI Clinic, Addenbrooke's Hospital University of Cambridge Cambridge UK

**Keywords:** membranous urethral length, pelvic floor contraction, post‐prostatectomy urinary incontinence, transperineal ultrasound, Valsalva maneuver

## Abstract

This study assessed the feasibility of dynamic transperineal ultrasound (TPUS) pre/post‐radical prostatectomy (RP). Ninety‐eight patients were scanned pre‐operatively and at four time‐points post‐operatively. TPUS was performed in 98 patients using an abdominal transducer at rest, during pelvic floor contraction (PFC) and Valsalva (VS) maneuver in supine and standing positions. Urodynamic evaluations included bladder neck angle at rest/PFC/VS, and degree of bladder neck movement. Pre‐operative and post‐operative measurements were technically feasible in >85% (supine) and >90% (standing) of patients. TPUS offers a reliable non‐invasive dynamic assessment of the pelvic floor post‐prostatectomy and may prove a useful adjunct for guiding exercises to preserve continence.

## Introduction

Prostate cancer is the second commonest male cancer and the fifth leading cause of carcinoma death worldwide.^1^ Since the 1980s, radical prostatectomy (RP) has become established as a key treatment option, recommended within European Association of Urology (EAU) guidelines^2^ for intermediate and high‐risk disease, with a subsequent 10‐year prostate cancer specific survive rate of around 99%.^3^ Nevertheless, the surgical approach is prone to potential short‐ and long‐term side‐effects including post‐prostatectomy urinary incontinence (PPUI) in 4 to 31%^4^ and sexual dysfunction in 14 to 90%,^5^, ^6^ which can impact on the psychosocial well‐being of patients, resulting in a significant reduction in the quality of life,^7^ and may impact heavily on healthcare resources.^8^

Due to the high prevalence of incontinence, understanding its pathophysiology has represented a challenge in clinical practice and in the research environment. The exact etiology along with pre‐ and peri‐operative risk factors for developing PPUI remains unknown, with theories including damage to the external sphincter detrusor, and underactivity and decreased bladder compliance.^9^ The widespread use of pre‐operative multiparametric magnetic resonance imaging (mp‐MRI)^10^ and recent advances in ultrasound technology have made imaging a feasible and attractive means of assessing pre‐ and post‐operative changes in both the prostatic bed and the pelvic floor. Although mp‐MRI has become the leading imaging technique for the detection, pre‐operative staging, and risk‐stratification of prostate cancer,^2^, ^11^, ^12^ only limited research has been undertaken for predictive features of PPUI in the pre‐operative setting.^13^

Transperineal ultrasound (TPUS) is a non‐invasive tool that has been widely used in the evaluation of female urinary incontinence and functional disorders of the pelvic floor,^14^, ^15^ however only limited studies have applied this technique for evaluation of the male pelvic floor.^16^, ^17^ TPUS has emerged as a functional imaging technique that could allow the dynamic evaluation of proximal urethral mobility and the pelvic floor in prostatectomy patients; however, its utility is yet to be studied in the combined pre and post‐operative settings. We hypothesize that TPUS during pelvic floor contraction (PFC) and the Valsalva (VS) manoeuver both in lying and standing position could help assess the anatomy and physiology of the pelvic floor, to help understand the mechanisms underlying the pathophysiology of PPUI. Therefore, the aim of this study was to demonstrate the technical feasibility of TPUS in patients before and after radical prostatectomy.

## Materials and Methods

### Study Participants

This prospective study entitled ProsPUR (The natural history of continence and incontinence post‐radical prostatectomy) was carried out with the approval of the local ethic committee (Cambridge University Hospitals NHS Foundation Trust, REC Ref: 11/EE/0086), with all participants signing written informed consent. Exclusion criteria included patients under the age of 18, patients who planned to have non‐local follow‐up, and patients having had prior surgical treatment for urinary incontinence. From December 2011 to March 2020, 98 patients underwent TPUS before and after robot assisted radical prostatectomy (RARP) as a treatment for localized prostate cancer. All prospectively enrolled patients were included, with the clinical and demographic characteristics, imaging features, surgical procedure and operative outcomes then retrospectively analyzed.

### Study Protocol

**Figure 1:**
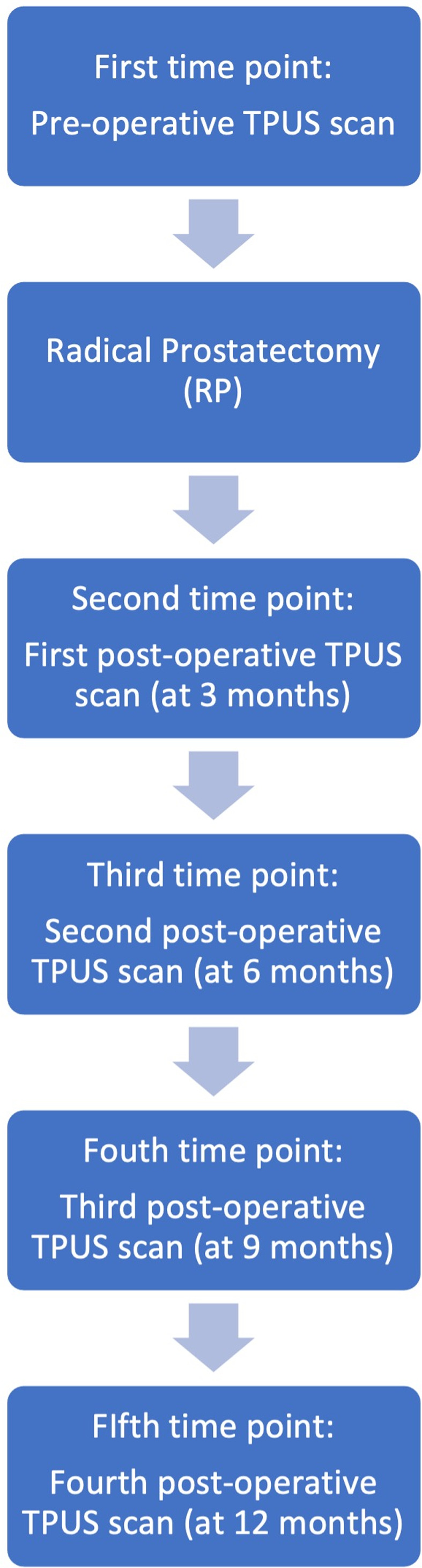
Study design.

Patients underwent five TPUS studies: one pre‐operatively, and four post‐operatively at 3, 6, 9, and 12 months, respectively (Figure 1). Ultrasound scans were performed by one of two experienced subspecialist uroradiology consultant radiologists (T.B., N.S.). Imaging was performed on a standard commercially available ultrasound machine (Toshiba Aplio XG, Canon Medical Systems, Ōtawara, Tochigi, Japan), using a low frequency curvilinear array abdominal transducer with a center frequency of 3.5 MHz (range 1.9–6 MHz). Static US images and real‐time videos were acquired in the dorsal recumbent/supine position and additionally in a standing position for the post‐operative scans. Images and real‐time videos were acquired by placing the probe on the perineal area, between scrotum and anus, in a sagittal orientation scanning across the pubic symphysis in order to visualize the bladder neck, the bladder and the urethra.^16^, ^17^ In both positions (lying and standing) the images were acquired under three different conditions: at rest, during PFC and during VS maneuver. The examinations took a maximum of 20 minutes. All patients enrolled in the study were clinically evaluated and completed the short‐form version of the International Consultation on Incontinence Questionnaire‐Urinary Incontinence (ICIQ‐UI SF); this questionnaire was used to measure continence outcomes, with continence strictly defined as an absence of urine leak.

### TPUS Measurements

**Figure 2:**
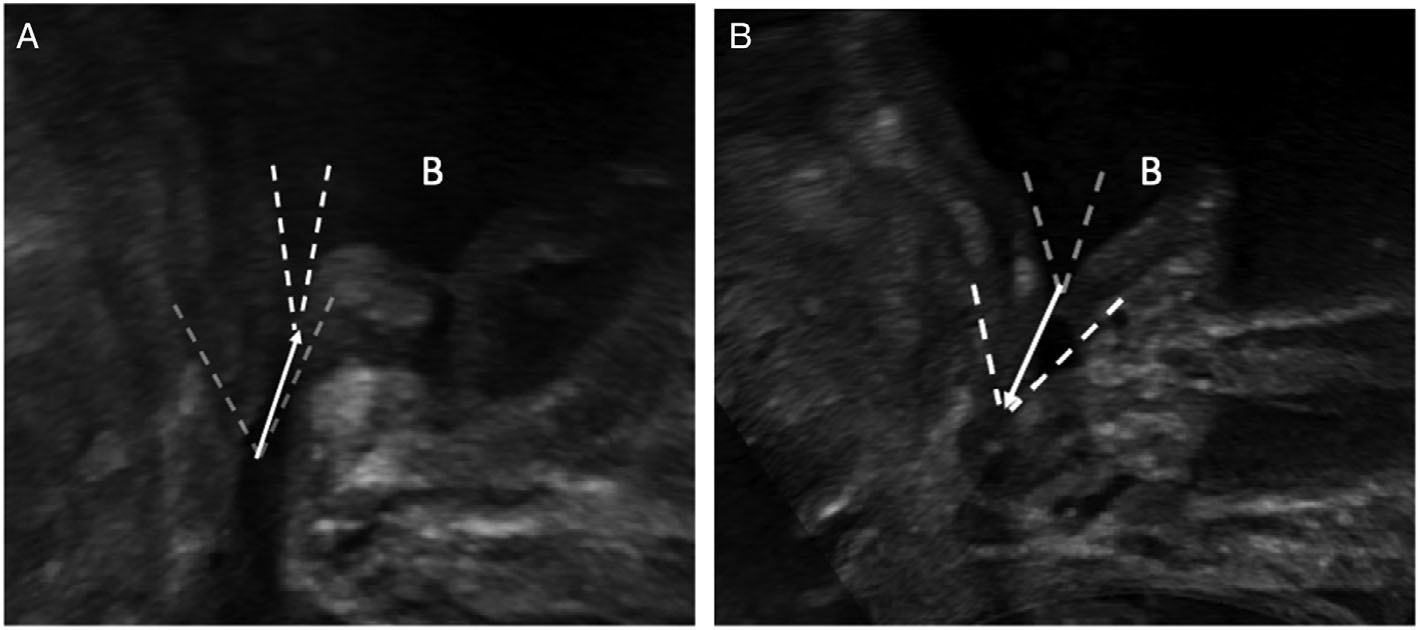
Superimposed images of the bladder neck (dashed lines) closing during PFC (**A**) and opening in VS (**B**) in the same patient. The resulting vector is indicated (arrow) for bladder ascent following PFC and bladder descend during VS. The letter B indicates the bladder. Images were acquired from the perineum, inferior to the prostate in the longitudinal/sagittal plane. The right‐hand side of the image is anterior and the left posterior to the patient.

The pre‐operative scan measurements included evaluation of prostatic intravesical protrusion, the membranous urethral length (MUL), the bladder neck angle (defined as the angle formed by the prostate and anterior bladder wall), at rest, after PFC and after VS manoeuvere. In addition, measurement of prostatic intravesical protrusion and MUL at TPUS were recorded and compared with the reference standard provided by the pre‐operative diagnostic mp‐MRI evaluation. For post‐operative scans, measurements were acquired for MUL, angle of the bladder neck at rest and following PFC/VS, and the degree of bladder neck ascent/descent during PFC and during VS manoeuver, with all measurements repeated with the patient standing (Table S1). Bladder neck ascent (during PFC) and descent (during VS) was assessed in mm, with the inferior aspect of the bladder neck as the baseline reference point, and the most superior (positive) displacement of the bladder neck for ascent and most inferior (descent), recorded relating to the *x* axis and *y* axis using a Cartesian plane^16^, ^17^ (Video S1). Based on this we calculated the resulting vector to evaluate the direction of the displacement (Figure 2). The static images and videos stored during the TPUS scan were independently analyzed in consensus by one of the US operators (T.B., N.S.) and one radiology resident (A.C.), with 4 years' experience in urological images.

### Statistics

To compare pre‐operative measurements acquired by the two techniques of mp‐MRI and TPUS, a Student's *t*‐test for paired data was carried out. *P* value <.05 was considered significant.

## Results

Ninety‐eight patients completed the study with a mean age of 62.5 years (range 48–73) and mean pre‐operative PSA of surgery of 10.1 ng/mL (range 2–57). Bilateral nerve sparing was performed in 30 patients, unilateral nerve sparing was performed in 47 patients (26 right side, 21 left side; Table 1). At pathological evaluation post‐surgery, 39 patients had T2 disease (9 with GS 6, 24 with GS 7, and 6 with GS 8), 53 patients had T3a (42 with GS 7, 4 with GS 8, and 7 with GS 9) and 6 patients had T3b disease (3 with GS 7, 2 with GS 8, and 1 with GS 9). Based on the ICIQ‐UI SF, at 1 year post‐prostatectomy, 52% of patients were incontinent, with the remaining 48% continent, defined as patients without leak of urine. All patients undergoing bilateral nerve sparing procedures recovered continence (19 had pT2 and 11 pT3a disease). The remaining 17 patients that recovered continence had unilateral nerve‐sparing surgery (11 had pT2 and 6 pT3a disease).

**Table 1:** Patient Clinical–Pathological Characteristics

Patient number	98
*Age (years)*	
Mean	62.5
Median	63.5
IQR 1,2,3	58.7, 63.5, 66
Range	48–73
*PSA (ng/mL)*	
Mean	10.1
Median	8.56
IQR 1, 2, 3	5.81, 8.56, 11.88
Range	2–57
*Biopsy Gleason Score*	
6 (3 + 3)	19
7 (3 + 4 and 4 + 3)	61 (47, 14)
8 (3 + 5, 4 + 4 and 5 + 3)	8 (4, 2, 2)
9 (4 + 5)	10
*Pathological stage*	
T2	39
T3 (T3a, T3b)	59 (53, 6)
*Pathological Gleason Score*	
6 (3 + 3)	9
7 (3 + 4 and 4 + 3)	69 (49, 20)
8 (3 + 5, 4 + 4 and 5 + 3)	12 (5, 4, 3)
9 (4 + 5)	8
*Nerve Sparing procedures*	
Not preformed	21
Unilateral (Right and Left)	47 (26, 21)
Bilateral	30

### Technical Note

During pelvic floor contraction (PFC), the prostate is visualized as being displaced superiorly in a linear fashion, and the urethra is compressed against the perineal body generating a compression in a dorsal direction,^18^ whereas during VS maneuver the prostate is observed to move in an inferior direction with translational rotation and posterior fanning. The presence of the prostate in the pre‐operative scans, typically with an enlarged transition zone (TZ) due to benign prostatic hyperplasia (BPH) made visualization of bladder neck more challenging. Prostatic calcification was not separately recorded but did not affect visualization in any individual patient. Conversely, the post‐operative absence of prostatic tissue and obliteration of the retropubic space brought the bladder neck closer to the transperineally probe and provided excellent views of the bladder neck, and often the urethral anastomotic sutures. Thus, post‐prostatectomy imaging allowed a clear evaluation of the MUL, the bladder neck opening during the VS maneuver and funneling. However, in some cases visualization of the urethra and bladder neck in the same plane was challenging, and fibrosis was difficult to objectively quantify, despite being more consistently visualized in the 9 to 12 month time‐points compared with the early post‐operative scans. Furthermore, the rectal ampulla was not consistently visualized due to presence of rectal gas, negating accurate measurement of the rectal angle.

### Objective Measurements

**Figure 3:**
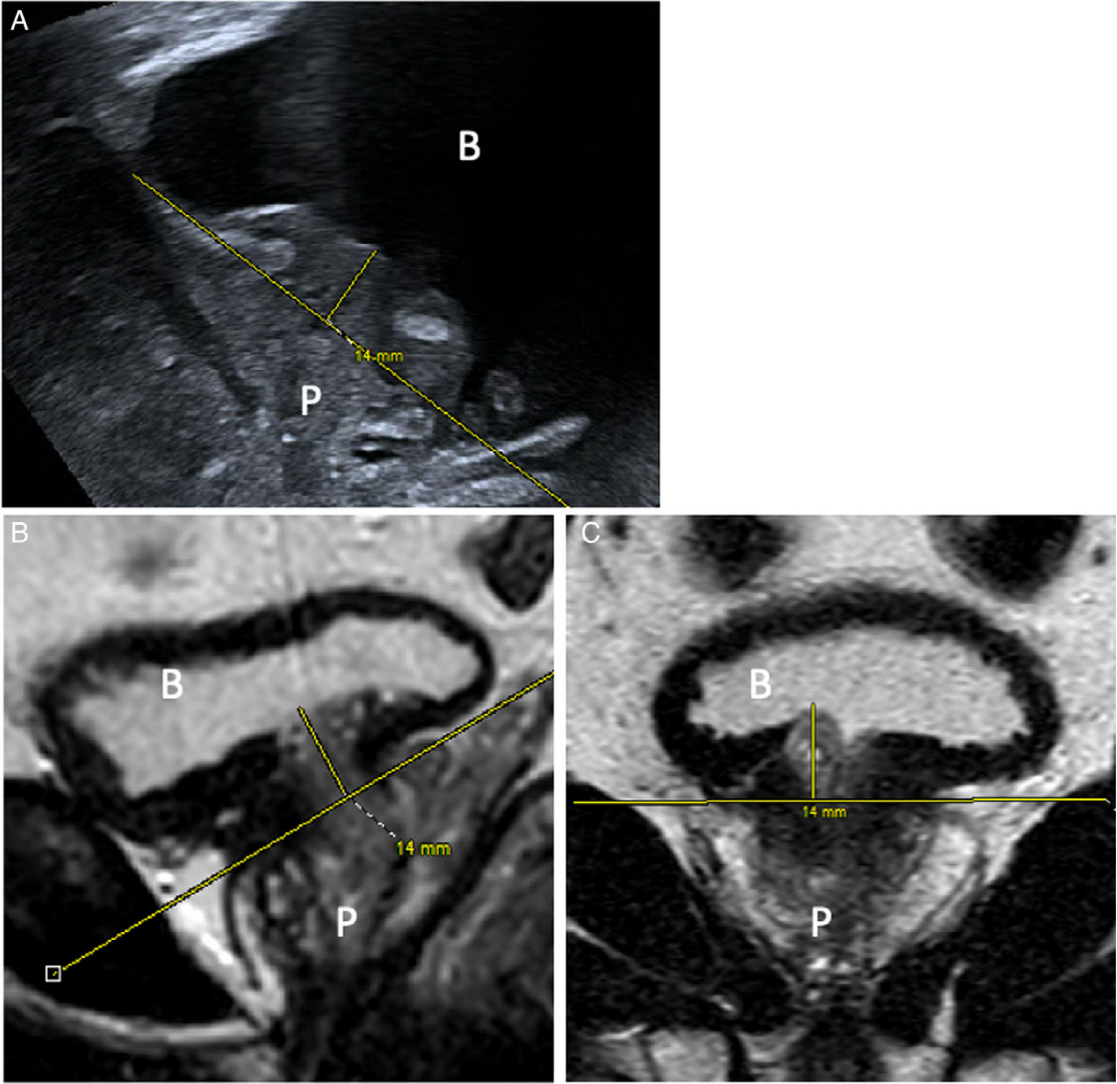
Pre‐operative TPUS image in the longitudinal plane (**A**) and mp‐MR images in sagittal (**B**) and coronal plane (**C**). The images show the intravesical prostate protrusion assessed by the two techniques. The letter “B” indicates the bladder and the “P” the prostate. In figure part A, the right‐hand side of the image is anterior and the left posterior to the patient.

Pre‐operative measurements for each of the proposed parameters were technically feasible in the majority of cases (range 88–95%), being highest for MUL and intravesicular bladder protrusion and lowest for bladder neck angle changes during VS maneuver (Table 2). The group average intravesical protrusion of the prostate was measured as 5.1 mm on TPUS, compared with 5.3 mm at MRI (Figure 3), the average MUL was 15.2 mm at TPUS evaluation, and 15.4 mm at MRI, with neither measurement showing significant difference (*P*‐values .14 and .76, respectively).

**Table 2:** Technical Feasibility of Parameters Assessed at Pre‐Operative Scanning

Measurements on pre‐operative scans	Feasibility (%)
Intravesical gland protrusion	95
Membranous urethral length (MUL)	92
Bladder neck angle before and after PFC and angle change	89
Ascendant during PFC (mm) along the *x* and *y* axis	89
Bladder neck angle before and after VS manoeuver and angle change	88
Descendant during VS manoeuver (mm) along the *x* and *y* axis	88

*Note*: All patients scanned in the supine position.

Abbreviations: PFC, pelvic floor contraction; VS, Valsalva.

**Figure 4:**
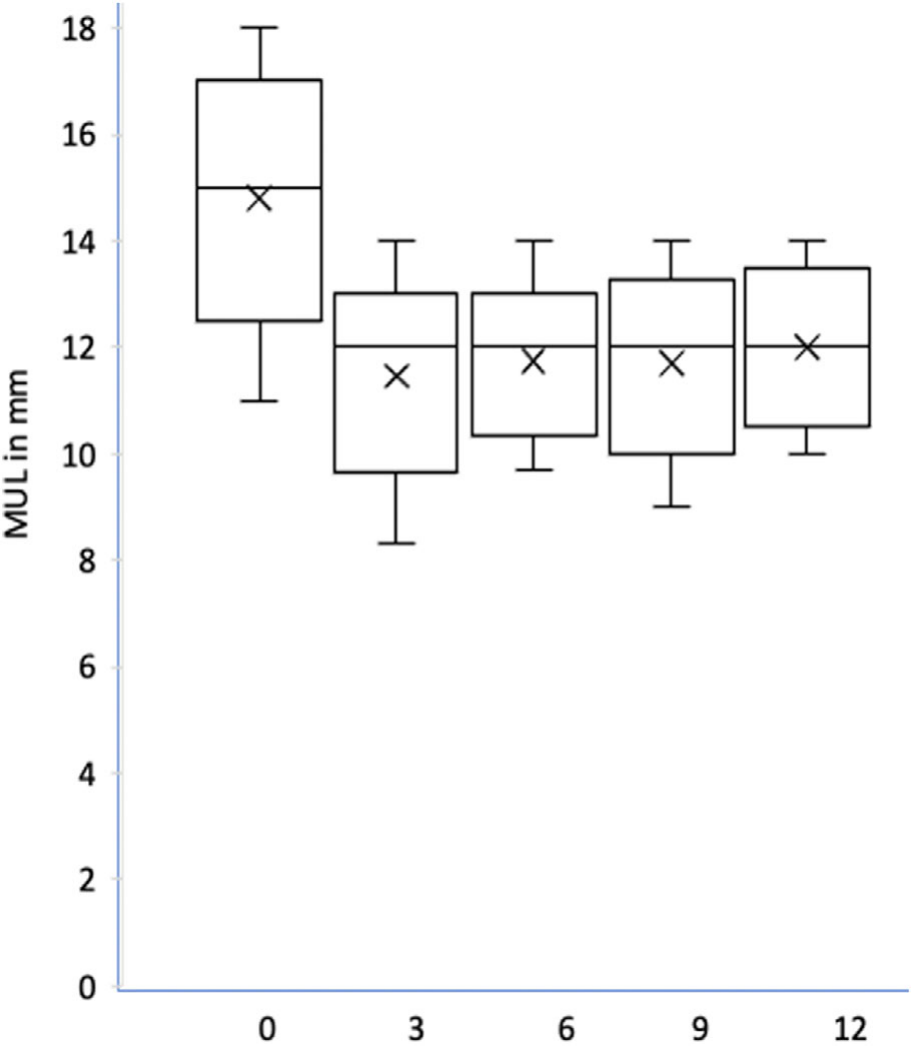
Box‐plot for distribution of average mean urethral length (MUL) over time. On the *y* axis the length of MUL in mm, on the *x* axis the different time points (pre‐operative in blue, 3 months post‐prostatectomy in orange, 6 months post‐prostatectomy in gray, 9 months post‐prostatectomy in yellow and 12 months post‐prostatectomy in light blue).

**Figure 5:**
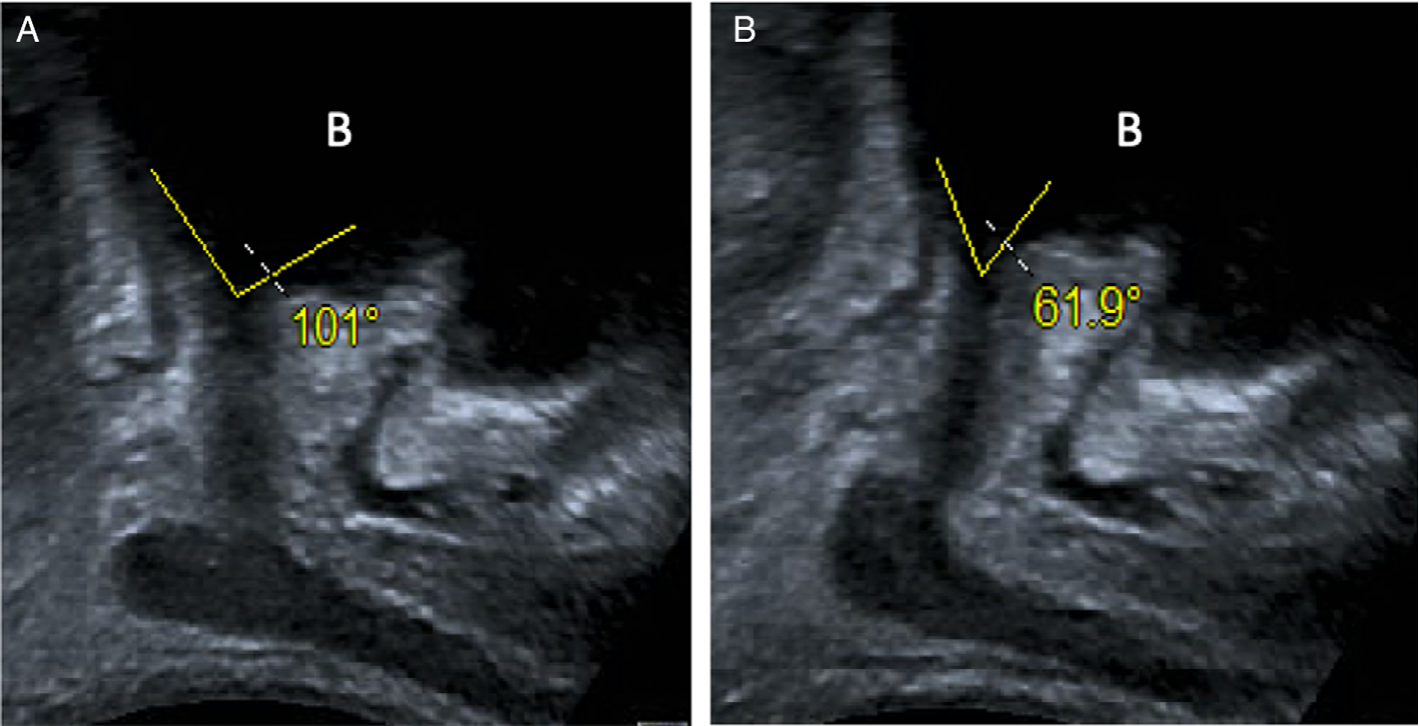
Post‐prostatectomy (12 months) TPUS images of a 62‐year‐old male at rest (**A**) and after PFC (**B**). Images show the elevation of urethra and the pelvic floor with a reduction of the angle of bladder neck. The letter B indicates the bladder. The right‐hand side of the image is anterior and the left posterior to the patient.

**Figure 6:**
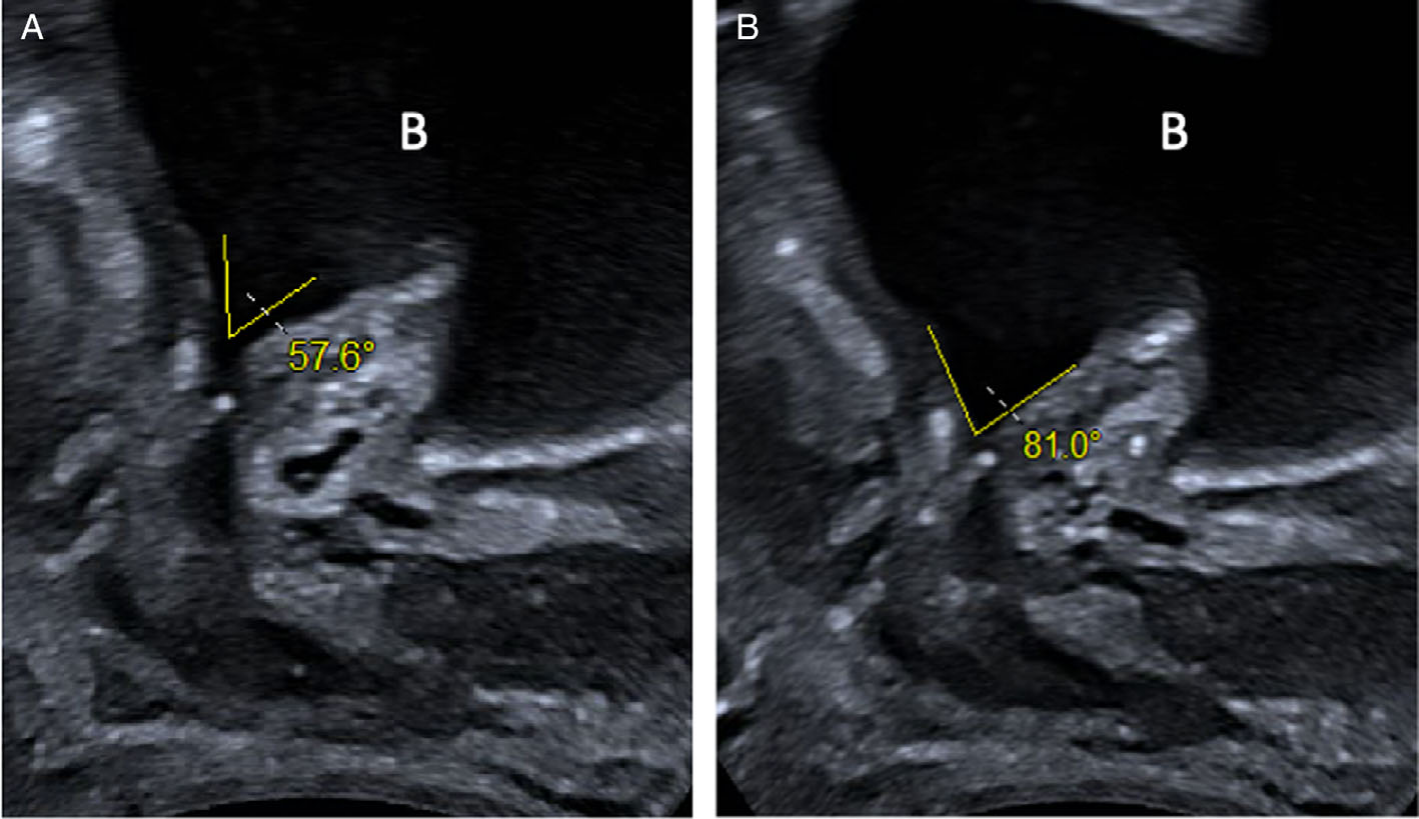
Post‐prostatectomy (12 months) TPUS images of a 62‐year‐old male at rest (**A**) and after VS maneuver (**B**). Images show the descendant of urethra and the pelvic floor with an increase of the angle of bladder neck. The letter B indicates the bladder. The right‐hand side of the image is anterior and the left posterior to the patient.

**Figure 7:**
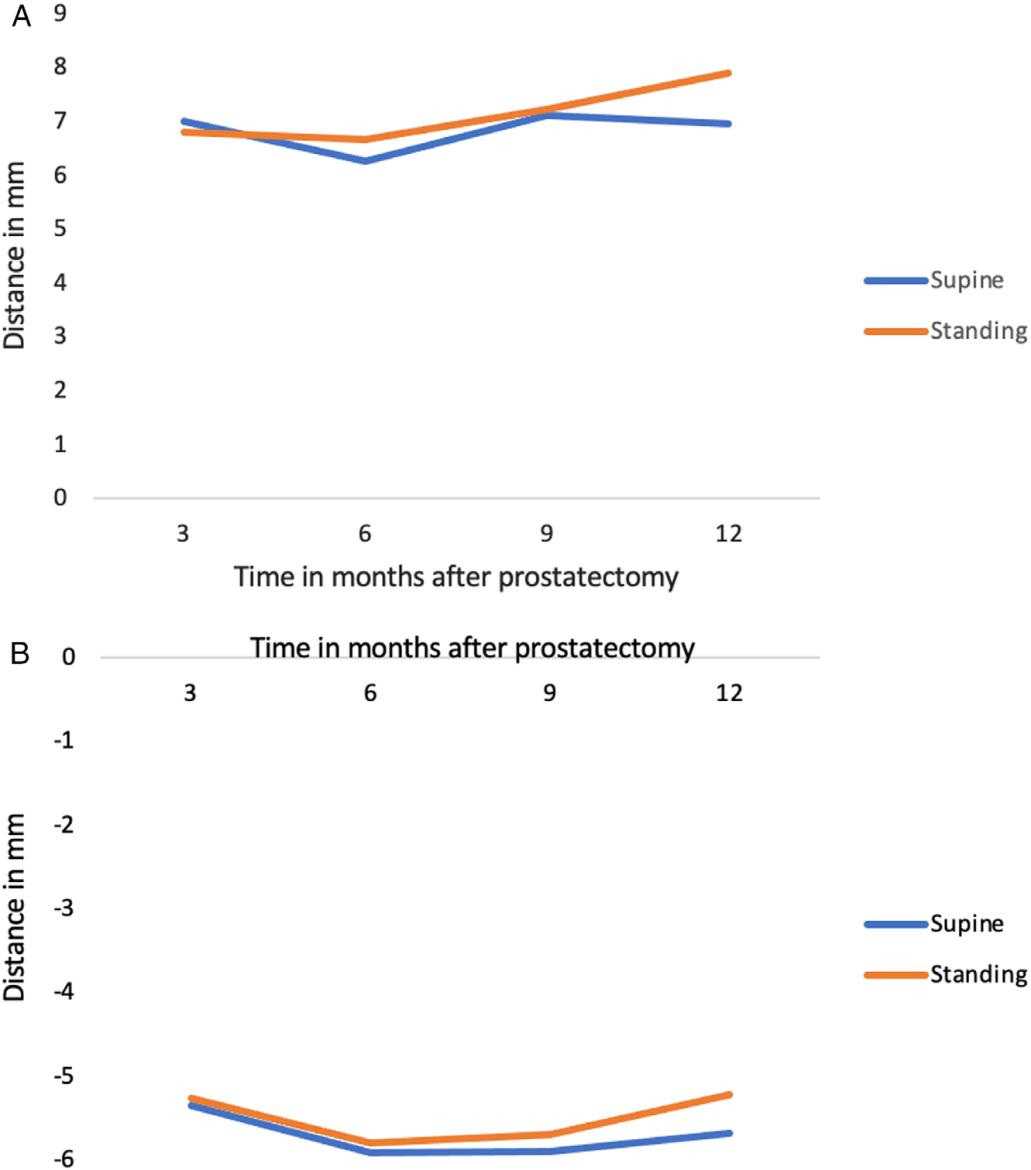
Average combined vector displacement during PFC (**A**) and in VS maneuver (**B**) in the supine and standing position.

**Figure 8:**
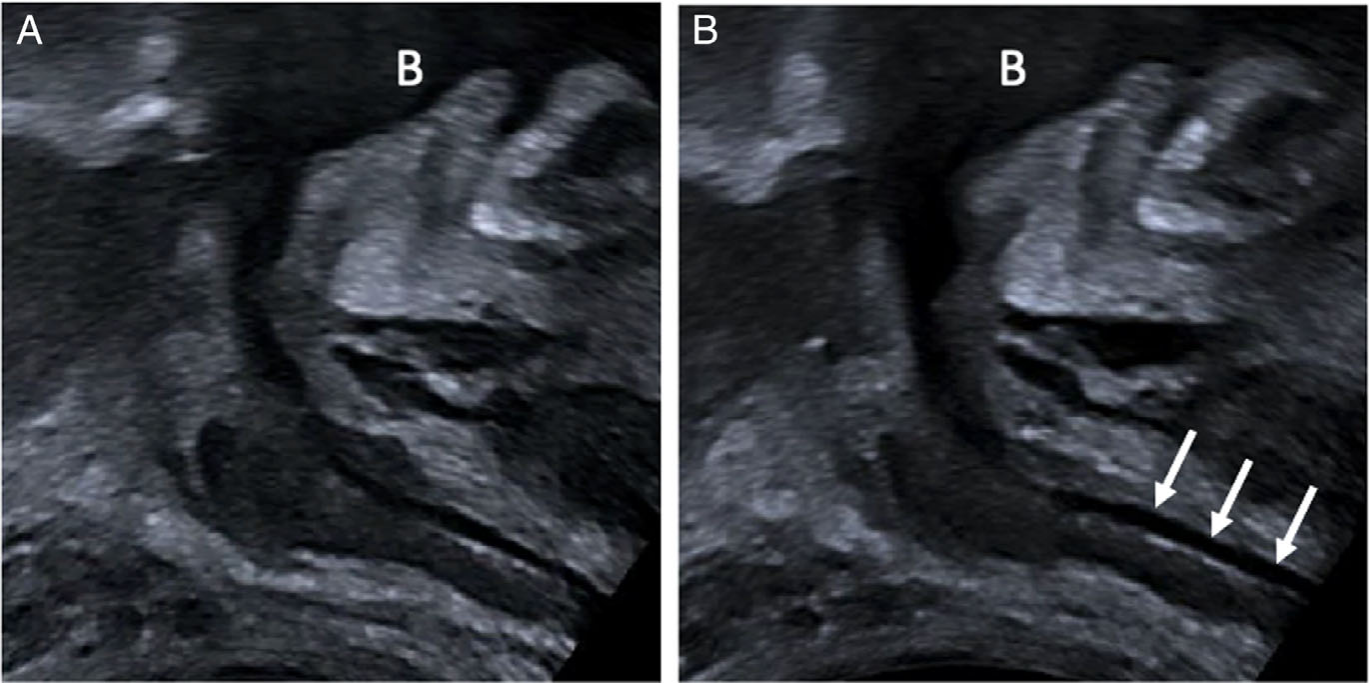
Post‐prostatectomy (12 months) TPUS images of a 68‐year‐old incontinent male at rest (**A**) and after VS maneuver (**B**). Images show the descendant of urethra and the pelvic floor; in these patients leak of urine defined as urine detected in the urethral tract was detected (with arrows). The letter B indicates the bladder. The right‐hand side of the image is anterior and the left posterior to the patient.

At post‐operative imaging, the technical feasibility of assessing parameters pre‐ and post‐PFC was high in both supine and standing positions, with a feasibility of 92.8 to 98.4% and 87.7 to 92.8%, respectively. The evaluation of parameters relating to VS maneuver, was also excellent both in supine and standing positions, with a feasibility of 92.8 to 97% and 87.7 to 93.5%, respectively; Table 3. Generally, the ability to evaluate PFC and VS‐related parameters was more technically challenging at 3 months post‐operatively compared with later time‐points, with 9 months post‐operatively representing the optimal time‐point. Additionally, technical feasibility for evaluating measurements relating to PFC and VS maneuver was superior for scanning in the supine position compared with standing for all of the considered time‐points (Table 3). As expected, there was a reduction in average MUL post‐operatively at 1 year (12 mm), compared with pre‐operatively (15.2 mm); Figure 4. Post‐operatively there was a general trend for an increasing degree of bladder ascent post PFC and increasing bladder descent post‐VS with increasing time post‐surgery (Figures 5, 6, 7 **)**. Furthermore, in a small number of patients (4/98), a bolus leakage of urine was noted to pass along the urethral tract during VS maneuver, consistent with stress incontinence (Figure 8; Video S2).

**Table 3:** Technical Feasibility of Parameters Assessed at Post‐Operative Scanning

Measurements on post‐operative scans	Feasibility at 3 month post‐operative scan		Feasibility at 6 month post‐operative scan		Feasibility at 9 month post‐operative scan		Feasibility at 12 month post‐operative scan	
Membranous urethral length (MUL)	95.4%		97%		98.4%		92.8%	
Bladder neck angle at rest, after PFC, and angle change	Supine	Standing	Supine	Standing	Supine	Standing	Supine	Standing
	94.5%	87.7%	97%	92.5%	98.4%	92%	92.8%	92.8%
Ascendant during PFC (mm) along the *x* and *y* axis	Supine	Standing	Supine	Standing	Supine	Standing	Supine	Standing
	94.5%	87.7%	97%	92.5%	98.4%	92%	92.8%	92.8%
Bladder neck angle at rest, after VS manoeuver, and angle change	Supine	Standing	Supine	Standing	Supine	Standing	Supine	Standing
	94.5%	87.7%	97%	91%	95%	93.5%	92.8%	92.8%
Descendant during VS manoeuver (mm) along the *x* and *y* axis	Supine	Standing	Supine	Standing	Supine	Standing	Supine	Standing
	94.5%	87.7%	97%	91%	95%	93.5%	92.8%	92.8%

*Note*: Patients scanned in both supine and standing positions.

Abbreviations: PFC, pelvic floor contraction; VS, Valsalva.

## Discussion

In this preliminary study, our aim was to describe the feasibility and the technique of TPUS in men before and after RP in patients with prostate cancer, with our study representing the first to incorporate both pre‐ and post‐operative ultrasound imaging for dynamic functional assessment in a surgical cohort. We report excellent technical feasibility for assessment of TPUS parameters before, during, and after PFC and VS maneuver and demonstrate a gradual increase in the degree of pelvic floor movements over a 12‐month post‐operative course.

Urinary incontinence remains a major side effect of radical prostatectomy (RP), impacting significantly on patients' quality of life and with implications for health care resources.^8^ It is well established that the first year after RP is crucial for the recovery of urinary continence,^19^ and imaging offers a potential means of identifying features that might predict and possibly prevent the development of PPUI. Multiparametric‐MRI is now established for the pre‐operative work‐up of prostate cancer, however, MRI only provides a static, morphological evaluation rather than a dynamic, functional one. TPUS is a technique which is non‐invasive, radiation‐free, cost‐effective and only moderately time‐consuming, and allows a functional assessment of the pelvic structures. However, despite having a pivotal role in the evaluation of female lower urinary tract,^20^ data is limited for the evaluation of PPUI in men, where a focus of the technique has been to guide biopsy sampling of the prostate in men who lack rectal access.^21^ To date, only a limited number of studies, with small cohorts have evaluated the role of TPUS in patients with prostate cancer undergoing surgery, the majority being post‐operative, with only one study additionally assessing the role of MUL pre‐ and post‐RP.^22^ Our study is the first to consider the dynamic, functional US assessment of patients before and after prostatectomy.

The results of this study demonstrate an excellent technical feasibility of TPUS at all considered time‐points, and particularly for post‐operative evaluations. The operator was consistently able to identify and evaluate anatomical landmarks and to assess their change during the two maneuvers. MUL is one of the few measurements to have an established predictive role for post‐operative recovery of continence,^13^ and we show MUL can be readily assessed with TPUS and with no significant difference in measurements compared with the reference standard of mpMRI. Likewise, pre‐operative intravesicular protrusion of the prostate has shown promise as a predictive measure^23^, ^24^ and could be assessed by TPUS with comparable results to MRI. These results show the potentially for TPUS as a pre‐operative technique for stratifying the risk of developing PPUI in a simple and rapid manner, and possibly to inform surgical choice, which may be particularly relevant in patients unable to undergo MRI for clinical reasons. However, dynamic evaluation of the functional mobility of the pelvic floor remains difficult pre‐operatively due to the presence of the prostate and variable degrees of BPH, which limits the assessment of urethral movement and funneling.

Conversely, for post‐operative scanning the probe is closer to the bladder neck in the absence of the prostate, allowing excellent functional assessment. Post‐operatively, we observed a general trend for increased dynamic movement of the pelvic floor over time with both PFC and VS maneuver, mirroring the known recovery period for continence in this patient population, and highlighting the progressive recovery of pelvic musculature, alongside functional improvement. The dynamic real‐time acquisition of TPUS^25^ enabled evaluation of bladder neck and urethral movement during PFC and of urethral funneling during VS manoeuver. This could prove useful for improved understanding of the pathophysiology of the pelvic floor structures and anatomical changes post‐RP in order to predict parameters related to the development of PPUI. Furthermore, the direct visualization of PFC by patients in the pre‐operative scan anecdotally helped in training men to perform contractions correctly as an exercise to preserve continence, and likewise the 3 month post‐operative scan enabled confirmation of such movement despite an altered post‐operative sensation.

The are some limitations to the current study. This was a technical evaluation and further assessments and correlations with clinical data and continence scores are essential in order to understand and quantify the value of this technique in clinical practice. The incidence of T3a disease (60%) was higher than some previously reported cohorts^26^, ^27^ and may have affected the prevalence of incontinence in our cohort. This is reflective of a specialist tertiary referral center and our established active surveillance program, with the majority of patients undergoing radical prostatectomy therefore harbor high intermediate‐ or high‐risk disease at greater volume and with an increased risk of extra‐capsular extension, negating nerve‐sparing surgery.^28^, ^29^ This is a single institution study and includes a relatively small number of patients in the cohort analysis; indeed, the technique would need to be tested using different ultrasound models, and by operators of differing experience and skill sets in order to ensure reproducibility and validity of the results.

## Conclusions

In conclusion, our results demonstrate the potential role of TPUS as a useful tool for guiding pelvic floor exercises in order to help to maintain continence after radical prostatectomy. The direct visual feedback provided could help patients learn the technique of PFC both pre‐operatively and during the recovery of pelvic floor mobility post‐operatively. TPUS is a dynamic technique that has potential to become an accurate, non‐invasive and cost‐effective modality in the evaluation of patients before and after prostatectomy.

## Supporting information


**Table S1** Parameters assessed by trans‐perineal ultrasound (TPUS) pre‐operatively and post‐operatively. All pre‐operative measurements performed with patients in the supine position; post‐operative measurements performed in both supine and standing positions. PFC, pelvic floor contraction; VS, Valsalva.

**Video S1** A, B. Post‐prostatectomy (12 months) TPUS of a 62‐year‐old male. The videos show the elevation of urethra and the pelvic floor with a reduction of the angle of bladder neck during PFC (A), and the descendant of urethra and the pelvic floor with an increase of the angle of bladder neck during VS manoeuver (B).

**Video S2** Post‐prostatectomy (12 months) TPUS of a 68‐year‐old incontinent male. The video shows the descendant of urethra and the pelvic floor; in these patients leak of urine defined as urine detected in the urethral tract was detected during VS manoeuver.
