# Supplementary material for: Transperineal Ultrasound Before and After Prostatectomy: Technical Approach and Description
Source: J Ultrasound Med. 2022 Jul 21;41(12):3125–35. doi: 10.1002/jum.16064 (PMC9796877; doi:10.1002/jum.16064)
Supplement: Supplementary file 1 — Table S1 Parameters assessed by trans‐perineal ultrasound (TPUS) pre‐operatively and post‐operatively. All pre‐operative measurements performed with patients in the supine position; post‐operative measurements performed in both supine and standing positions. PFC, pelvic floor contraction; VS, Valsalva. [file JUM-41-3125-s003.docx]

**Supplementary Materials**

**Supplementary Table 1**

**Parameters assessed by trans-perineal ultrasound (TPUS) pre-operatively and post-operatively.** All pre-operative measurements performed with patients in the supine position; post-operative measurements performed in both supine and standing positions. PFC = pelvic floor contraction, VS = Valsalva.

| **Measurements at the preoperative scan** | **Measurements at the postoperative scans** |
| --- | --- |
| Intravesical gland protrusion |  |
| Membranous urethral length (MUL) | Membranous urethral length (MUL) in the supine position alone |
| Bladder neck angle before and after PFC | Bladder neck angle before and after PFC |
| Change in angle (difference between the angle at rest and afterPFC) | Change in angle (difference between the angle at rest and after PFC) |
| Ascendant during PFC (mm) along the x and y axis | Ascendant during PFC (mm) along the x and y axis |
| Bladder neck angle before and after VS manoeuver | Bladder neck angle before and after VS manoeuver |
| Change in angle (difference between the angle at rest and after PFC) | Change in angle (difference between the angle at rest and after PFC) |
| Descendant during VS manoeuver (mm) along the x and y axis | Descendant during VS manoeuver (mm) along the x and y axis |

**Supplementary Video 1 A, B**

Post-prostatectomy (12 months) TPUS of a 62-year-old male. The videos show the elevation of urethra and the pelvic floor with a reduction of the angle of bladder neck during PFC (A), and the descendant of urethra and the pelvic floor with an increase of the angle of bladder neck during VS manoeuver (B).

**Supplementary Video 2**

Post-prostatectomy (12 months) TPUS of a 68-year-old incontinent male. The video shows the descendant of urethra and the pelvic floor; in these patients leak of urine defined as urine detected in the urethral tract was detected during VS manoeuver.
